# Immunological Deregulation in Classic Hodgkin Lymphoma

**DOI:** 10.4084/MJHID.2014.039

**Published:** 2014-06-01

**Authors:** Alessandra Romano, Calogero Vetro, Giovanni Caocci, Marianna Greco, Nunziatina Laura Parrinello, Francesco Di Raimondo, Giorgio La Nasa

**Affiliations:** 1 Division of Haematology, Azienda Ospedaliera “Policlinico-Vittorio Emanuele”, University of Catania. Via Citelli 6, 95124 Catania, Italy; 2 Hematology Unit, Department of Medical Sciences “Mario Aresu,” University of Cagliari, Italy

## Abstract

Classic Hodgkin Lymphoma (cHL) has a unique histology since only a few neoplastic cells are surrounded by inflammatory accessory cells that in the last years have emerged as crucial players in sustaining the course of disease. In addition, recent studies suggest that the abnormal activity of these inflammatory cells (such as deregulation in regulatory T cells signaling, expansion of myeloid derived suppressor cells, HLA-G signaling and natural killer cells dysfunction) may have prognostic significance. This review is focused on summarizing recent advanced in immunological defects in cHL with translational implications.

## Introduction

In the classic Hodgkin’s lymphoma (cHL) microenvironment, a few neoplastic cells - Hodgkin and Reed-Stemberg (HRS) cells- grow in a contest of a tissue rich in immune system cells, including fibroblasts, eosinophils, lymphocytes, histiocytes, neutrophils and monocytes.^1^ These immune system cells are unable of mounting effective anti-tumor immune responses and, on the contrary, even stimulate and promote the growth of HRS cells.

The strong correlation between classic HL (cHL) and Epstein-Barr virus (EBV) infection strengthens the hypothesis that alterations in the mechanisms involved in viral clearance (antigen presentation, innate natural killer cell-dependent immune response) may influence the onset of cHL.

For its peculiar histology cHLis an extremely interesting study model for the assessment of immunogenetic factors that may confer susceptibility to tumours or, alternatively, facilitate tumour immune escape mechanisms.

After the demonstration of the prognostic significance of Interim-2-[18F]Fluoro-2-deoxy-D-glucose Positron Emission Tomography (PET-2) performed in the mid of chemotherapy, the role of accessory cells in cHL has been evaluated with increased interest. In fact, it has been demonstrated that PET-2 positivity is mainly due to the Fluoro-2-deoxy-D-glucose uptake by the accessory cells rather than the HRS cells.^2^

Inflammation-related accessory cells can be indirectly evaluated in the peripheral blood as well: several reports investigated the prognostic impact of the ALC/AMC-DX ratio, obtained by dividing the absolute lymphocyte count (ALC) over the absolute monocytes count (AMC) from the complete blood count, as a surrogate of host immune homeostasis and tumour-associated macrophages (TAM) respectively, with contrasting results.^3^, ^4^

This review is focused on the novel advances about the role of myeloid and lymphoid subsets involved in sustaining HRS and favouring immune-escape.

## NK Dysregulation

Natural killer (NK) cells represent a key component of the innate immune system against cancer.

Together with NK cells, a subset of CD1d-restricted Natural Killer-T cells (NKT) exhibits direct anti-tumour activity and enhances cytotoxicity of NK and CD8+ T cells. NKT cells are distinct lymphocyte population characterized by the expression of CD3 and CD56 and an invariant T-cell receptor (TCR) formed by the Ja18-Va24 and Vb11 rearrangements specific for glycosphingolipids presented by the non-classical MHC Class-I molecule CD1d.^5^

A common immune escape strategy of HRS cells is to down-regulate the expression of human leukocyte antigen (HLA) -A,-B and -C (classic: MHC Ia) and to modify the expression of HLA-G and E (no classical: MHC Ib), as seen in about 20% and 80% of primary cases of EBV+ and EBV- cHL, respectively. However, because communication through MHC Ia-specific inhibitory receptors on NK cells is lacking, downregulation of MHC Ia generally leads to the activation of NK cells.^6^

The paucity of NK cells in the reactive infiltrate of cHL and the systemic NK cell deficiency observed in cHL patients prompted further investigation into the immune-modulatory mechanisms of NK receptors such as the NKG2D activating receptor of the C-type lectin superfamily, killer immunoglobulin-like receptors (KIRs), immunoglobulin-like transcript 2 (ILT2) inhibitory receptors, immunoglobulin-like transcript 4 (ILT4) and the NKG2A inhibitory receptor. New evidences continue to emerge that a reduced activity of NK cells may be related to the prevalence of inhibitory over activating KIR genes.^7^

Therapeutic strategies aimed at interfering with the crosstalk between HRS cells and their cellular partners have inspired the development of new immunotherapies targeting different cellular components of the microenvironment.

NKG2D receptor and the group of natural cytotoxicity receptors (NCRs) (NKp46, NKp44, and NKp30) are regarded as the major NK cell receptors in tumour defence. Immune surveillance via NKG2D and the corresponding ligands seems to be particularly effective in the early stages of tumour growth.^8^

However, tumour cells develop escape mechanisms to evade NK cell surveillance and NKG2D-ligand interaction, which obviously results in either immune activation (tumour clearance) or immune silencing (tumour evasion).

Silencing of NKG2D during tumour progression results from the persistent exposure of ligands expressed on the surface of target cells. Moreover, tumour cells release ligands into the environment by shedding. The soluble molecules not only block NKG2D, but also induce the internalization and degradation of the receptor.^9^

Plasma levels of soluble ligands correlate with disease progression in many haematological and solid tumours. Former studies on NK cell function in HL have shown that peripheral NK cells from patients with HL are functionally inactive. The observed NK cell dysfunction correlates to elevated serum levels for ligands engaging NKG2D (MICA) and NKp30 (BAG6/BAT3). Low levels of the membranous NKG2D-ligands, i.e. MHC class I related chain-A (MIC-A) and UL16 binding protein 3 (ULBP3), on HRS cells presents another way to escape from cytotoxic T-cell responses. These low levels are the result of proteolytic cleavage of the NKG2D-ligands by ERp5 and a disintegrin and metalloproteinase domain-containing protein 10 (ADAM10) produced by HRS cells and mesenchymal stromal cells. Additionally, T-cells in cHL tissue have lower NKG2D receptor expression levels as compared to T-cells in normal lymph nodes, due to TGF-β produced by the mesenchymal stromal and HRS cells, which blocks IL-15-induced expression of NKG2D receptor on cytotoxic T-cells. Thus, the anti-tumour activity of CD8+ T-cells is blocked by lack of membrane NKG2D-ligands, release of soluble NKG2D-ligands and reduced NKG2D receptor levels on effector T-cells.^10^

Immunotherapeutic strategies targeting NK cells are promising because NK cell cytotoxicity could be restored in vitro and patients using a novel human antibody construct specifically designed for the treatment of cHL and other CD30-expressing malignancies. In a previous study, a tetravalent bispecific antibody construct (AFM13) was used to target CD30 on HRS cells with two of its binding sites, whereas the activating receptor CD16A on NK cells (CD30xCD16A, AFM13) was targeted by the other two binding sites, thereby selectively cross-linking tumour and NK cells.^11^

Also, epigenetic modifications have been implicated in the malignant phenotype of HRS cells. In this context, the histone-deacetylase (HDAC) inhibitor LBH589 (panobinostat) was shown to be clinically effective. LBH589 modulates the crosstalk of lymphocytes with HL cell lines. More specifically, LBH589 induces cell death, autophagy, and an increase of major histocompatibility complex (MHC) class I chain-related genes molecules (MICA/B); that act as key ligands for NK cell receptors, and also favourably modulates the cytokine network and lymphocyte activity in the HL microenvironment.^12^ Studies of innovative therapies based on the immune system of HL patients treated with chemo/radiotherapy and targeting NK cells rather than T cells are, therefore, extremely promising.

## Lymphoid Impairment

Lymphoid anergy is well-known in the biology and pathogenesis of cHL and T-cell homing is central in determining the immunological regulation of HRS growth and survival.^13^,^14^ The lymphoid infiltrate in HL is different from the aspecific one detectable in reactive lymphoid hyperplasia (RLH), since the CD3+/CD20+ ratio is greater in HL than in RLH,^15^ with augmented CD4+CD25+ infiltrate.^15^,^16^

Whole-tissue RNA analysis evaluated the specific microenvironment characteristics of HL, discovering that a great release of cytokines is present alongside suppressed expression of apoptotic genes and augmented expression of cell-cycle regulatory enzymes. HRS cells genotyping analysis showed a global suppression of principal tumor suppression pathways, including Rb-p16INK4, p27KIP1, p53 and an increased expression of components of G1-CDK checkpoint.^17^,^18^ The neoplastic HRS themselves create a favorable microenvironment for their survival and growth regulating inflammatory infiltrate. In vitro studies on KM-H2 HRS cell-line demonstrated that HRS cells are able to induce CD4+CD25+ regulatory T-cells (Treg),^19^ whose function is to inhibit the cytotoxic effects of CD8+ T-cells, the so called cytotoxic lymphocytes (CTL).^16^,^19^,^20^

Main factors involved in lymphocyte migration into the tumor milieu include CCL20,^21^ CCL5/Rantes, IL-7, CCL17 and CCL22.^22^ CXCR3, CXCR4, CXCL13 and CCR7, and adhesion molecules including CD62 ligand (CD62L), are greatly expressed on T-cells of cHL patients.^23^ HRS cells are also able to express chemokine receptors useful to T-cell migration into tumor milieu, such as CXCL12 (receptor of CXCR4) and CXCR5 (receptor of CXCL13).^24^

T-regs accumulate into the tumor milieu thanks to the surface expression of CCR4, the receptor for “thymus and activation regulated chemokine” (TARC/CCL17), a factor greatly secreted by HRS cells.^25^ T cells stimulated with TARC acquire a regulatory function, able to silence the cytotoxic activity of CTL.^26^ Conversely, CTL are not influenced by TARC since they lack the surface expression of CCR4.^27^

Once migrated into the tumor mass, lymphocytes are addressed toward Th2 and T-reg differentiation (in particular, a Tr1 phenotype)^28^ acquiring the ability (together with HRS cells) to produce and secrete TGF-β and IL-10, able to suppress CTL function. Thus, T-regs regulate the production of IL-2 and limit CTL activation,^23^ while Th2 cells induce the expression of several cyclins and cyclin-dependent kinases 29 and of antiapoptotic markers, such as Bcl-Xl and Mcl1,^30^ with overexpression of STAT3 in HRS cells, activation of cyclin D1 and Bclx expression, and a down-regulation of STAT1, a tumor suppressor factor.^17^,^18^,^31^

CTL are further silenced through the CD95-CD95L and PD1-PD1L cell-to-cell contact between HRS cells and CTL.^32^–^37^ Additionally, the production of galectin-1, tissue inhibitor of metalloproteinase1 (TIMP1), and prostaglandin E2 (PGE2) by HRS cells inhibits CTL function with impairment of the IFN-γ production and induction of the Th2 and T-reg expansion.^29^,^38^–^40^

## Myeloid Derived Suppressor Cells

Recent investigations suggest that a subset of myeloid cells, the so called “myeloid-derived suppressor cells” (MDSCs) are the progenitors of tumour associated macrophages,^41^–^43^ that are considered among the most important and emerging prognostic factors in HL.^44^

MDSC have been identified in solid and haematological cancers as a heterogeneous population of immature and mature cells of myeloid origin able of leading the tumour escape from immune-surveillance, through depletion of arginin and cystein due to the high expression level of arginase (Arg-1), nytrosylation of T-cell receptor, reactive oxygen species (ROS) release, thus being responsible of cancer progression as recently reviewed.^41^,^45^,^46^ The term suppressive refers to the peculiar ability to elicit T-cell anergy thanks to the above-mentioned biochemical pathways.

In mice two distinctive mononuclear (Ly6G-, low “side-scattered light”-SSC) and polymorphonuclear (Ly6G+, high SSC) tumour-induced MDSC have been identified, while the phenotype in humans is still controversial.^47^ Overall, current evidence suggests a complex alteration of myeloid cell differentiation and function in human cancer patients that involves polymorphonuclear^48^ and monocytic cells.^49^ A frequently used combination of markers for human MDSC includes CD33^+^/CD11b^+^/HLA-DR^−^ and CD14^+^/HLA-DR^low^ to define monocytic MDSC (mo-MDSC), CD66b^+^/CD15^+^/CD11b^+^/CD14^−^ or CD11b^+^/CD13^+^/CD15^+^/CD14^−^/HLA-DR^−^/Lin^−^ for the identification of granulocytic MDSC (N-MDSC) and CD13^+^/CD14^−^/CD34^+^/HLA-DR^−^ for the immature subset MDSC (im-MDSC).

T cell dysfunction induced by MDSC can reflect the recruitment of inflammatory cells and favour the aberrant MDSC production, setting up a pathological loop.^45^

Our group hypothesized that the amount of MDSC in peripheral blood of cHL-patients may reflect the complexity of cytokine and cell-cell contacts of the pathologic neoplastic microenvironment and that the myeloid cellular impairment could represent a prognostic factor in cHL at diagnosis. Preliminary data from our single-centre small series of 60 newly diagnosed cHL-patients identify an increase of the absolute count of im-MDSC, N-MDSC and mo-MDSC in peripheral blood at diagnosis (Romano, manuscript submitted).

Progression free survival of patients carrying high levels of MDSC at baseline was poor. In multivariate analysis, im-MDSC high levels were an independent predictor of inferior outcome despite a PET-2 based risk adapted treatment (Romano, manuscript submitted).

## Soluble Factors

### TARC

The CC chemokine ligand 17 (CCL17), also well-known as Thymus and Activation-Regulated Chemokine (TARC), is a member of the CC chemokine group constitutively expressed in the thymus. TARC is produced by monocyte-derived dendritic cells and binds specifically to the CC chemokine receptor 4 (CCR4), mainly expressed on T-regs and Th2 cells of the reactive infiltrate.^50^ TARC is considered a Th2-type chemokine because CCR4-expressing T cells mainly produce interleukin (IL)-4.

In more than 90% of cases HL lymph-nodes have a positive TARC staining in HRS cells detected by immunohistochemistry, with high specificity for HL since the low/absent expression in anaplastic large cell lymphoma or T-cell-rich B-cell lymphoma.

In about 85% of patients, TARC is detectable and elevated in serum at diagnosis before treatment.^51^,^52^ Pre-treatment serum TARC levels correlate with stage of disease, erythrocyte sedimentation rate, leukocyte and lymphocyte counts,^51^,^52^ pre-treatment metabolic tumor volume, as measured by quantification of 2-[18F]fluoro-2-deoxyglucose positron emission tomography images, and to treatment response.^53^

### HLA-G

The expression of non-classical Human leukocyte antigen G (HLA-G) is another strategy adopted by HRS cells to evade immune defence and to create protected niches where they grow and expand. HLA-G is expressed on HRS cells in more than 50% of HL patients and is associated with lack of HLA class I expression and tumour cell EBV status. HLA-G is a non-classical major histocompatibility complex (MHC) Class I product with limited sequence variability. The HLA-G gene generates seven isoforms by alternative splicing encoding HLA-G1, -G2, -G3, and -G4 membrane-bound protein isoforms and HLA-G5, -G6, and -G7 soluble protein isoforms.

The properties of soluble and membrane-bound HLA-G proteins are different, but in general, both are regarded as being immunosuppressive.^54^

HLA-G is a tolerogenic molecule which inhibits cytolysis mediated by NK cells or T lymphocytes, induces T cell apoptosis and blocks transendothelial migration of NK cells and these roles are performed upon binding the KIR2DL4 and the ILT2 and ILT4 ligands.^55^

It is known that antigen-presenting cells expressing membranous HLA-G can induce regulatory T cells in freshly isolated peripheral blood mononuclear cells, in vitro and that soluble HLA-G induces regulatory T cells in an antigen non-specific manner.^56^

The latter can inhibit CTL responses and is present in the cHL reactive infiltrate.^57^ Alterations in HLA-G antigen expression and function are often induced in tumours and are likely to be mediated by various microenvironmental factors. Interestingly, immunohistochemistry and flow cytometry evaluations have shown expression of HLA-G protein in a large number of solid and some hematopoietic malignancies, e.g. cutaneous lymphomas, chronic lymphocytic leukemia (CLL) and diffuse large B-cell lymphoma. In CLL, some B-cell, T-cell non-Hodgkin’s lymphomas, and leukemia, plasma levels of soluble HLA-G are increased. Soluble HLA-G serum and plasma levels have been useful markers for the prediction of some of these malignancies.^57^

A population-based study showed that protein expression of HLA-G by HRS cells is common at primary cHL diagnosis and that this expression is associated with lack of EBV and absence of cell surface expression of MHC Ia on HRS cells. The consequence of HLA-G expression or sHLA-G is an escape from T and NK cell-mediated recognition. Thus, alterations of non-classical and classical HLA class I antigens and components of the antigen-processing pathway provide tumour cells with different mechanisms to inactivate immune responses resulting in tumour growth and evasion from host immune surveillance.^7^

### sCD163

Recently, an increasing interest has been focused on the amount of CD68+ tumor associated macrophages (TAM) infiltration.^44^ The amount of TAM is strongly associated with shortened survival in cHL, correlated with likelihood of relapse after autologous stem cell transplantation and outperformed the current International Prognostic Score (IPS) for disease-specific survival.^44^

The functional characterization of TAM is still to be performed and, possibly, differences in survival among patients could be explained by the macrophages M1/M2 binary on which these cells differentiate, or by the histological signature of myeloid derived immunosuppression. Increasing evidences in mammary tumor model suggests that the most immunosuppressive activity is played by TAM derived from circulating MDSC, but it is still an open question.

The antigen CD163 is physiologically expressed on the macrophage surface, and it is currently investigated as an additional marker of macrophage infiltration in HL microenvironment, since the lack of reproducibility of CD68 staining. An increased infiltration of CD163/CD68 (M2 macrophages) was associated to poor outcome, with a rise in treatment-related deaths and poor event-free survival, disease-specific survival and overall survival.^58^ Recently, the circulating fraction of CD163 in serum (s-CD163) has been evaluated in patients at diagnosis and relapse, showing not being inferior to TARC to identify patients with poor outcome.^59^

## Conclusions

Despite high initial cure rate, almost 20% of cHL patients fails front line therapy and have a median overall survival less than three years. Increasing evidences suggest that failure to conventional therapy is not only due to the intrinsic resistance of HRS cell but accessory cells and the so called microenvironment play an important role. The network and the relationship between the HRS and accessory cells are not fully elucidated, but several studies have highlighted new pathways that currently are under investigation as prognostic markers, including HLA-G, s-CD163 and MDSC. In addition, new immunological target are emerging in cHL microenvironment, including NK, NKT and MDSC that in the future could be treated with specific drugs. Actually, the introduction of targeted immunotherapy has induced an increasing interest about the prognostic implication of the microenvironment and its manipulation with drugs able to elicit an immune response.
